# Lactase bacteria in intestinal mucosa are associated with diarrhea caused by high-fat and high-protein diet

**DOI:** 10.1186/s12866-022-02647-2

**Published:** 2022-09-28

**Authors:** Kang Zhou, Maijiao Peng, Na Deng, Zhoujin Tan, Nenqun Xiao

**Affiliations:** 1 College of Pharmacy, Hunan University of Chinese Medicine, Changsha, Hunan China; 2 College of Chinese Medicine, Hunan University of Chinese Medicine, Changsha, Hunan China; 3 Hunan Key Laboratory of TCM Prescription and Syndromes Translational Medicine, Changsha, Hunan China; 4 College of Medicine, Hunan University of Chinese Medicine, Changsha, Hunan China

**Keywords:** High-fat and high-protein diet, Gene diversity, Lactase bacteria, Diarrhea, Intestinal mucosa

## Abstract

**Background:**

Excessive fat and protein in food can cause diarrhea by disturbing the intestinal microecology. Lactase is a functional enzyme strongly associated with diarrhea, while lactase bacteria in the intestine are an important source of microbial lactase. Therefore, we reconnoiter the relationship between diarrhea induced by a high-fat and high-protein diet (HFHPD) and intestinal mucosal lactase bacteria from the perspective of functional genes.

**Result:**

Operational Taxonomic Units (OTUs) were 23 and 31 in the normal group (NM) and model group (MD), respectively, and 11 of these were identical. The Chao1 and Observed specie indexes in the MD were higher than those in the NM, but this was not significant (*P* > 0.05). Meanwhile, the Principal coordinate analysis (PCoA) and Adonis test showed that the community structures of lactase bacteria in NM and MD were significantly different (*P* < 0.05). In taxonomic composition, lactase bacteria on the intestinal mucosa were sourced from Actinobacteria and Proteobacteria. Where Actinobacteria were higher in NM, and Proteobacteria were higher in MD. At the genus level, *Bifidobacterium* was the dominant genus (over 90% of the total). Compared to NM, the abundance of *Bifidobacterium* were lower in MD, while MD added sources for lactase bacteria of *Rhizobium*, *Amycolatopsis*, and *Cedecea*.

**Conclusions:**

Our data demonstrate that HFHPD altered the community structure of lactase bacteria in the intestinal mucosa, decreased the abundance of the critical lactase bacteria, and promoted the occurrence of diarrhea.

## Background

The intestinal microbiota is a complex ecosystem consisting of approximately 10^14^ microorganisms in the intestine, which regulate immunity, maintain the intestinal mucosal barrier, and assist the host in digesting food [1]. The intestinal mucosa is the first contact surface between substances and the intestine. It plays a vital role in nutrient absorption and serves as an important immune barrier to block harmful substances from entering the body. Intestinal microbiota and intestinal mucosal immunity have a close relationship. Intestinal microbiota can promote intestinal mucosal lymphoid tissue maturation [2], enhance mucosal B cell responses [3], and secret substances beneficial to mucosal epithelial cells to shape intestinal mucosal immune barrier function [4]. Simultaneously, intestinal mucosal function's integrity also contributes to intestinal microbiota stability and the growth of mucosal commensal bacteria.

With the westernization of the modern diet, people's intake of dietary fiber-rich foods such as coarse grains and cereals gradually decreases, while the intake of high-fat and high-protein foods is steadily increasing [5]. HFHPD is not a healthy dietary pattern, in which a high-fat diet can raise endotoxin levels, increases intestinal mucosal permeability, and upregulates the expression of pro-inflammatory and pro-tumor factors, in turn promoting the occurrence of gastrointestinal tumors [6–8]. Moderate protein intake is beneficial, but a high-protein diet might produce the opposite result. Protein that exceeds the body's digestive threshold will ferment in the intestine, producing amines, H_2_S, and ammonia. These toxic metabolite products can damage the intestinal mucosa, reduce intestinal immunity and increase the number of pathogenic bacteria such as Coliforms, *Streptococcus,* and *Bacillus* [9, 10]. Jiayuan et al. [11] also found that HFHPD decreased the abundance of the probiotic *Lactobacillus* and increased the abundance of the opportunistic pathogen *Helicobacter* in the intestinal mucosa of mice, which in turn triggered diarrhea. Another study found that HFHPD promoted the colonization of *Clostridioides difficile* (*C. difficile*) in the intestine of mice [12]. *C. difficile* is highly associated with diarrhea, and its production of toxins can lead to cell death and the development of diarrhea [13]. Notably, diarrhea by induced HFHPD may also be associated with decreased enzyme activity. The digestion of food requires enzymatic support, and the rich enzyme genes in the intestinal microbiota compensate for the lack of enzymes secreted by humans themselves [14]. Correspondingly, food may also affect enzyme activity by shifting the enzyme-producing flora, as we found in our previous study that a significant decrease in the activity of several intestinal enzymes in model mice with diarrhea by induced HFHPD, including lactase, which is associated with diarrhea [15].

Lactase is a digestive enzyme distributed in the intestinal mucosa and contents, also known as β-galactosidase. If lactase is deficient, the residual lactose will be processed by intestinal bacteria such as Bacteroides, Clostridia, etc. These bacteria will break down and ferment lactose rapidly, producing large amounts of short-chain fatty acids and gas, which raises the osmotic pressure in the intestinal lumen, causing diarrhea [16]. Current research has found that functional dyspepsia, diarrheal irritable bowel syndrome, and AAD are associated with lactase deficiency [17–19]. Some lactase gene-containing bacteria (i.e. lactase bacteria) in the intestine are essential providers of lactase, such as *Bifidobacterium* sp., *Bacillus* sp., and *Escherichia coli* [20]. Depending on the gene encoding, the lactase activity produced by bacteria varies greatly. It can be distinguished as high activity, low activity, and no activity [21, 22]. This indicated the lactase activity of microbial origin is well plasticity. In the previous study, we found that AAD reduced lactase activity and changed the community diversity and structure of lactase bacteria to different degrees [19, 23]. Simultaneously, the alleviation of AAD by the Chinese medicinal compound Qi Wei Bai Zhu San and probiotic *Debaryomyces hansenii* was related to promoting the growth of key lactase bacteria [24–27]. Further, Gingold-Belfer et al. [28] found that supplementation with probiotics with β-galactosidase activity has improved symptoms in patients with lactose intolerance. This means that the change of lactase bacteria had a high correlation with lactase activity, and the change of lactase bacteria by HFHPD might be one of the critical factors contributing to diarrhea. However, there were few studies on the relationship between HFHPD and intestinal mucosal lactase bacteria.

Based on the microbial lactase gene perspective, this study aimed to investigate the community characteristics of lactase bacteria in the intestinal mucosa of mice with diarrhea induced by HFHPD. And provide an experimental basis for the mechanism study of diarrhea induced by improper diet. In addition, by the current disease state, some specific bacteria can be screened for developing corresponding microbial therapies [29]. Therefore, our research on lactase bacteria will also provide directions for developing relevant functional agents.

## Results

### General behavioral observation of animals

The fur of NM mice was smooth and flat with good mental status. The stool was moderately soft and hard, slightly deformed when picked up with tweezers. MD mice had loose fur, decreased glossiness, reduced activity, and squinting. The stool was soft, easily deformed and stuck to the tweezers, more than half of it was thick paste, adhering to the tail and perianal area.

### Effect of HFHPD on the OTU count of intestinal mucosal lactase bacteria

**Fig. 1:**
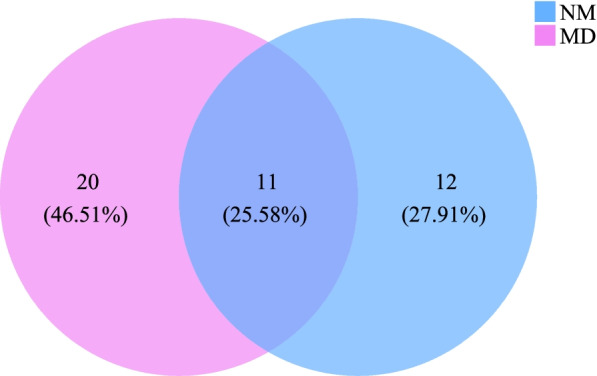
Venn diagram of intestinal mucosal lactase bacterial OTU

993,571 effective sequences were obtained at the end of sequencing, of which 931,184 were high-quality sequences, accounting for 93.73%. Venn diagrams visually represent the percentage of common and unique parts between different groups. As shown in Fig. 1, the OTUs unique to the MD 20, accounting for 46.51%. The OTUs unique to the NM 12, accounting for 27.91%. The common OTUs were 11, accounting for 25.58%. It is suggested that HFHPD increased the number of taxonomic units of lactase bacteria in the intestinal mucosa of mice.

### Alpha diversity analysis

**Fig. 2:**
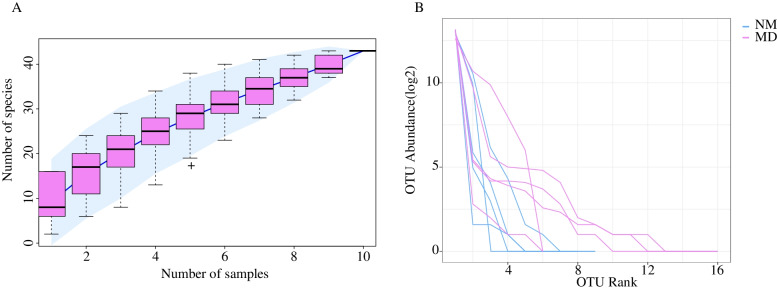
**A** Species cumulative curves. **B** Rank abundance curves. NM: normal group; MD: model group

The species accumulation curve can measure and predict the increase of species richness in the community with the expansion of sample amount, which reflects whether the current sequencing depth is sufficient to reflect the genetic diversity of the existing samples. The species accumulation curve in Fig. 2(A) shows that the increase of species has leveled off and can be used for species composition analysis of the colony. On the other hand, the rank abundance curve can visually reflect the number of high abundances and rare OTUs in the sample community. The steeper the curve in the horizontal direction, the lower the evenness and richness in the sample community. In Fig. 2(B), compared to MD, the NM was steeper in the horizontal direction and flatter in the vertical direction. This indicated that the evenness and richness of lactase bacteria in MD are relatively higher.

**Fig. 3:**
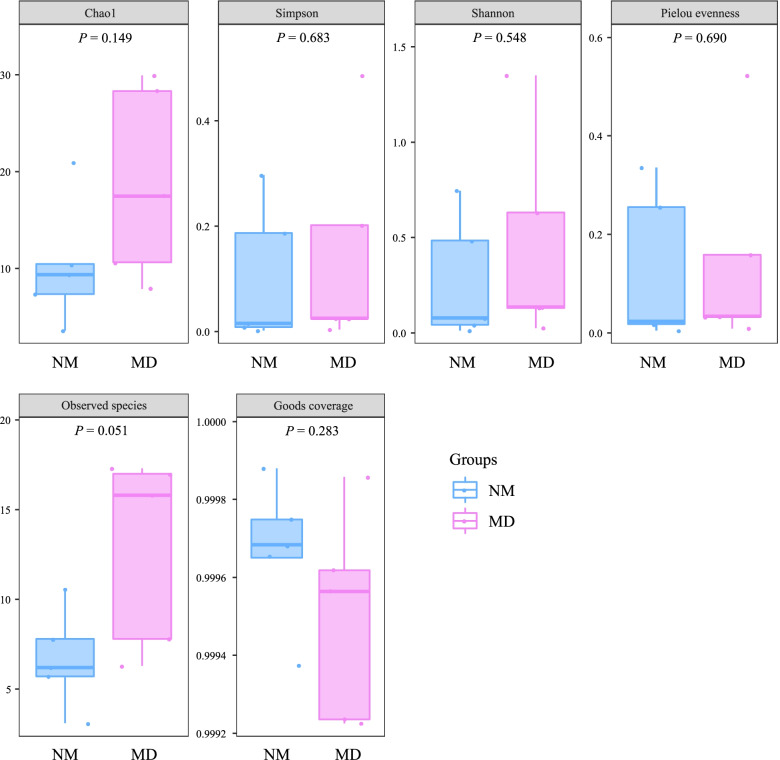
Alpha diversity index of lactase bacteria in mice intestinal mucosa. The numbers under the diversity index label are the *P* values of independent t-test or Wilcoxon rank-sum test. NM: normal group; MD: model group

The diversity and richness of each sample in a microbial community can be evaluated by Alpha diversity. The larger Chao1 and Observed species (observed OTUs) indices indicate the higher species richness of the community [30]. Simpson is a common index for evaluating community diversity. The higher the value of the Simpson index, the higher the diversity of the community, and the Shannon index integrates the richness and evenness of the community on this basis [31, 32]. The pielou evenness index is used to evaluate the evenness of the community, with higher values indicating a more even community [33]. The Goods coverage index indicates the species coverage, with higher values indicating a smaller proportion of undetected species in the sample [34]. As seen in Fig. 3, the Goods coverage index was over 99.9% in both the NM and MD, indicating that the number of species covered by sequencing was high enough. Among the remaining indices, the Shannon, Pielou evenness, and Simpson indices of NM and MD were almost not different, while Chao1 and Observed species indices could be relatively higher in MD. This was similar to the results of OTU analysis and species accumulation curve, but the Alpha diversity indices were not statistically significant (*P* > 0.05). The above results suggest that HFHPD has an increasing effect on the richness of the lactase bacterial community in the intestinal mucosal.

### Beta diversity analysis

**Fig. 4:**
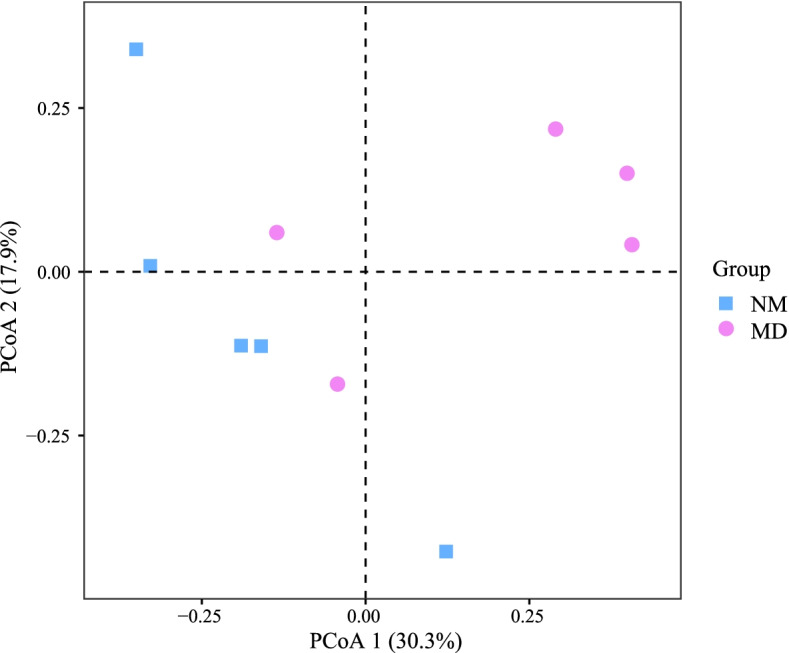
PCoA of community composition of lactase bacteria in mice intestinal mucosa. PCoA plots were constructed using the Jaccard distance matrix. NM: normal group; MD: model group

The differences in community structure between samples can be quantified by dimensionality reduction. PCoA can expand the sample distance matrix in the low-dimensional space after projection and retain the distance relationship of the original samples to the maximum extent, which is more suitable for ecological data characteristics than principal component analysis [35]. Figure 4 shows the PCoA analysis based on Jaccard distance (plotting with R language v4.1.1), in which PCoA one explained 30.3% of variation and PCoA two 17.9% explained. The samples of the MD were mainly concentrated in the first quadrant, and the samples of the NM were primarily gathered in the second and third quadrants, and the two groups could be relatively distinguished. Simultaneously, the Adonis test indicated the community composition was significantly different between the NM and MD (Jaccard, R^2^ = 0.18, *P* < 0.05). It indicates that HFHPD changed the compositional structure of lactase bacteria in the intestinal mucosa of mice.

### Taxonomic composition and abundance of lactase bacterial in the intestinal mucosa

**Fig. 5:**
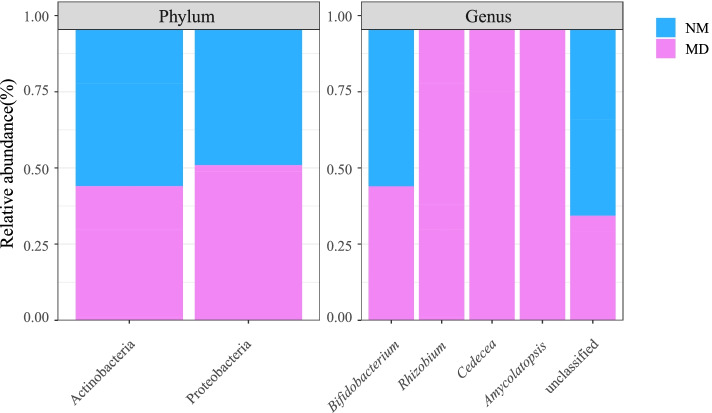
Taxonomic compositions of lactase bacteria in the intestinal mucosa. NM: normal group; MD: model group

From Table 1, the intestinal mucosal lactase bacteria sources were Proteobacteria and Actinobacteria. Lactase dominant bacteria were *Bifidobacterium* of Actinobacteria, and the percentage of chief sources in both groups was above 90%. By comparison (Fig. 5), it was found that the abundance of Actinobacteria and *Bifidobacterium* were higher in the NM, while the abundance of Proteobacteria, *Rhizobium*, *Amycolatopsis*, and *Cedecea* were higher in the MD, and *Rhizobium*, *Amycolatopsis*, and *Cedecea* were exclusive to the MD. In conclusion, community composition suggests that HFHPD increased the number of taxonomic species of lactase bacteria while decreasing the abundance of dominant lactase bacteria.

**Table 1:** Taxonomic composition of lactase bacterial in the intestinal mucosa

	NM	MD
Actinobacteria	0.998129 ± 0.002709	0.980132 ± 0.039450
Proteobacteria	0.001871 ± 0.002709	0.019868 ± 0.039450
*Bifidobacterium*	0.995978 ± 0.003845	0.979687 ± 0.039235
*Rhizobium*	0.000000 ± 0.000000	0.001306 ± 0.002612
*Amycolatopsis*	0.000000 ± 0.000000	0.000006 ± 0.000008
*Cedecea*	0.000000 ± 0.000000	0.000010 ± 0.000008
unclassified	0.004022 ± 0.003845	0.018991 ± 0.036632

*NM* normal group, *MD* model group

## Discussion

The intestinal microbiota can participate in host food metabolism by secreting various enzymes, and providing energy for the growth and development of the organism. Some of these enzyme-producing strains can also influence the physiological and pathological status of the organism by secreting differential enzymes or regulating the expression of enzyme activities [14]. For example, Nada et al. [36] reported that the potency of *Bifidobacterium longum* and *Lactobacillus acidophilus* decreased the expression of COX2 enzyme in human gastric cancer cell lines. Li et al. [37] found that *Streptococcus thermophilus* can inhibit colorectal tumorigenesis by secreting lactase. Meanwhile, the distribution characteristics of microbial enzyme genes have good potential to reflect the functional diversity of microbial communities, and can be used as biomarkers to judge the health of the body [19, 38].

In Alpha diversity, the richness of lactase bacteria in MD increased after HFHPD intervention (*P* > 0.05). An association between high-fat diet or high-protein diet and intestinal microbiota Alpha diversity has been reported, but this association is not consistent. For example, Alpha diversity increased [39, 40], decreased [41, 42], or changed slightly [43] after high-fat diet or high-protein diet intervention. Our previous studies [11] have shown that HFHPD can significantly increase the diversity and abundance of intestinal mucosal bacteria, but it is not clear whether the combination of high-fat diet and high-protein diet is the cause. We hypothesized that adequate HFHPD in the short term caused adaptive changes in the microbiota, but was not significant for the lactase bacterial diversity in the intestinal mucosa.

The PCoA results indicated that the community structure of lactase bacteria in intestinal mucosa was changed after intervention with HFHPD (*P* < 0.05). The lactase bacteria in the intestinal mucosal were sourced from Proteobacteria and Actinobacteria, of which the abundance of Actinobacteria accounted for more than 97%. The MD increased the abundance of Proteobacteria and decreased the abundance of Actinobacteria. Studies have shown that Proteobacteria may be a potential factor in various diseases, and increased numbers of members belonging to this phylum have been found in patients with obesity, nonalcoholic fatty liver hepatitis, intestinal colitis, and atherosclerosis [44–46]. Actinobacteria are absolute players in maintaining intestinal homeostasis, the most representative being *Bifidobacterium *[47]. *Bifidobacterium* is the dominant bacteria and probiotics in human body, with beneficial effects such as modulating immune function, reducing the inflammatory response, and producing short-chain fatty acids [48]. Moreover, *Bifidobacterium* is also a major strain for lactase production. Both *Bifidobacterium animalis* and *Bifidobacterium longum* can be used as probiotic additives with sound alleviating effects on lactose intolerance [49]. The decrease in *Bifidobacterium* promotes increased intestinal permeability, leading to lipopolysaccharides' translocation and triggering chronic inflammatory diseases [49, 50]. We found that after HFHPD intervention, the abundance of *Bifidobacterium* in the MD was decreased, and increased exclusive lactase bacteria, including *Rhizobium*, *Amycolatopsis*, and *Cedecea*. *Rhizobium* is a bacterium mainly distributed in soil, but may also cause bacterial infections [51]. *Amycolatopsis* is an important source of several bioactive natural products with good antibiotic-producing potential [52]. *Cedecea* is a rare conditionally pathogenic bacterium that usually proliferates in immunocompromised patients [53]. Lactase activity is determined by gene expression, but not all lactase enzymes express activity. Presently, researches related to lactase by *Rhizobium*, *Amycolatopsis*, and *Cedecea* are few, which means that it is unlikely that these non-dominant bacteria have a relatively superior lactase production capacity compared to *Bifidobacterium*.

In addition, the intestinal ecology varies significantly from region to region. Therefore, microorganisms in different intestinal areas are relatively different in their composition, function, and response to external substances. For example, the intestinal mucosal microbiota is more sensitive to repeated stress-related diarrhea than the intestinal content microbiota, and more sensitive to dietary factors than the fecal microbiota [42, 54]. Furthermore, the body lactase could be derived from the villi of intestinal mucosal epithelial cells, and the integrity of the intestinal mucosal is an important guarantee for the lactase to play the function of digestion of nutrients. A variety of pathological conditions leading to small intestinal damage, including celiac disease, Crohn's disease, and small bowel bacterial overgrowth, can lead to decreased expression of lactase, from causing transient lactose intolerance to diarrhea. [55] Meanwhile, multiple studies have demonstrated the negative effects of a high-fat or high-protein diet on the intestinal mucosa [6, 8, 9, 43]. This suggests that HFHPD on one hand may cause a disruption of the lactase distribution environment or expression conditions by damaging the intestinal mucosa. On the other hand, HFHPD could decrease the abundance of critical lactase bacteria, and increase the abundance of lactase bacteria with low enzyme producing activity, which in turn affects lactase activity and intestinal microbiota homeostasis, leading to the development of diarrhea.

## Conclusion

The results showed that HFHPD altered the community structure of lactase bacteria, decreased the abundance of crucial lactase bacteria, and increased the abundance of strains with low lactase production capacity, such as *Rhizobium*. This affected lactase activity, promoted the occurrence of diarrhea, and may have injured the intestinal mucosal barrier. In addition, as this is a pilot study to explore the relationship between HFHPD and lactase bacteria, it has several limitations. For example, the sample size was small, and the results were only based on the lactase gene of intestinal mucosal bacteria, without excluding the interference of other microbial enzyme genes (such as lipase and protease). In future studies, the above results need to be further validated by enlarging the sample size, mixing lactose in the diet, or supplementing it with the corresponding lactase bacteria.

## Methods

### Animals

Ten SPF Kunming male mice weighing 18–22 g were purchased from Hunan Sleika Jingda Experimental Animal Co. Ltd (SCXK (Xiang) 2019–0004) and housed in the Animal Experiment Center of Hunan University of Chinese Medicine (room temperature 23℃ ~ 25℃, relative humidity 50% ~ 70%).

### Diets

The conventional feed and bedding were purchased from Hunan Sleika Jingda Experimental Animal Co (protein: 20%, fat: 4%). The high-fat and high-protein feed was made by mixing milk powder (Nestle, 30% protein and 20% fat), soybean milk flour (Huiyi, 33% protein and 18% fat), flour (Huiyi, 13% protein and 2% fat) and meat pine (AnhuiLizheng, 30% protein and 25% fat). Specific production method: the above ingredients are mixed in the ratio of 1:2:2:1, add the appropriate amount of water and stirred into a paste, shaped into a cylinder similar in size to the ordinary feed, and placed in the oven at 70 ℃ drying, 72 h after taking out that is obtained. Vegetable oil (Arawan, 59.0% soybean oil, 21.0% rapeseed oil, 10.0% sunflower seed, 3.0% corn oil, 3.0% peanut oil, 3.0% rice oil, 0.6% sesame oil and 0.4% linseed oil). All the above materials contain no lactose.

### Reagents

Tris-saturated phenol–chloroform-isoamyl alcohol (25:24:1), lysozyme, proteinase K, chloroform, isoamyl alcohol, acetone, and TE buffer were purchased from Beijing Dingguo Biotechnology Co. Ltd. 10% sodium dodecyl sulfate (SDS), 0.1 mol/L phosphate buffer solution (PBS) buffer, 5 mol/L NaCl, chloroform-isoamyl alcohol (24:1), cetyl trimethyl ammonium bromide (CTAB)/NaCl, 3 mol/L sodium acetate and 70% anhydrous ethanol, were prepared in the laboratory.

### Animal experimental process

**Fig. 6:**
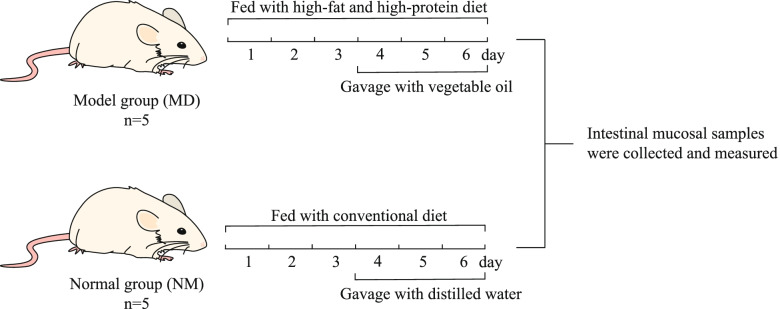
Experimental flow

After 10 mice were acclimatized and fed for 2 days, the mice were divided into the normal group (NM) and model group (MD) according to a random number table, and no blinding was done. Each group had 5 mice each, 5 in 1 cage. The modeling method was referred to in the reference [16], and the procedure is shown in Fig. 6. The mice in the MD were fed with HFHPD, and gavage with vegetable oil (0.4 mL/time, 2 times/day) was started on day 4. The mice in the NM were fed with conventional diet, and gavage with distilled water (equal dose and frequency) was started on day 4. If the mice showed diarrhea, the modeling was successful.

### Intestinal mucosa collection from mice [56]

After 6 days of HFHPD intervention, all mice were euthanized using rapid cervical dislocation to reduce suffering. Cervical dislocation was performed by an experienced person. After ensuring the death of all mice, the intestinal tracts from the jejunum to ileum were removed under aseptic conditions, cut open with surgical scissors, rinsed in physiological saline to remove the attached contents, and placed on sterile filter paper to remove water. The intestinal mucosa was scraped with a slide on the weighing paper. The scraped intestinal mucosa was put into a sterile tube, weighed, and stored at a low temperature.

### DNA extraction

DNA extraction method reference [19]. The intestinal mucosa was homogenized in 0.1 mol/L PBS and centrifuged at 200 × g for 2 min. After the supernatants were washed twice with PBS, they were centrifuged again at 10,000 × g for 8 min. The precipitate was washed three times with PBS, twice with acetone, and finally resuspended in TE buffer. 45 μL of TE buffer, 20 μL of lysozyme, and 5 μL of protease K were added to 500 μL of bacterial suspension and incubated at 37 ℃ for 30 min, then mixed with 30μL10% SDS and incubated for 40 min, vortex every 10 min. Then 80 μL of CTAB/NaCl and 100 μL of 5 mol/l NaCl were mixed, and that mixture was vortexed at 65 ℃ for 10 min. To the sample add an equal volume of Tris-saturated phenol–chloroform-isoamyl alcohol (25:24:1) and centrifuge at 10,000 × g for 3 min. After the supernatant was centrifuged at 10,000 g using an equal volume of chloroform-isoamyl alcohol (24:1) for 3 min, the supernatant was centrifuged again under the same conditions as chloroform-isoamyl alcohol (24: 1). Transfer the obtained supernatant into a new germ-free tube, add 0.1 times the amount of 3 mol/L sodium acetate and twice the amount of anhydrous ethanol, and precipitate at -20 ℃ for 12 h. The samples were centrifuged at 10,000 × g for 3 min. The formed precipitate was washed with 70% ethanol, dried, and dissolved in 50 L TE buffer.

### PCR amplification and sequencing

Bacterial lactase gene amplification reference [57], upstream primer: 5′-TRRGCAACGAATACGGSTG-3′ and downstream primer: 5′-ACCATGAARTTSGTGGTSARCGG-3′. PCR amplification system: Q5 high-fidelity DNA polymerase 0.25 μL, 5 × Reaction Buffer 5 μL, 5 × High GC Buffer 5 μL, dNTP (10 mM) 0.5 μL, template DNA1 μL, upstream primer (10 μM) 1 μL, downstream primer (10 μM) 1 μL and ultrapure water 11.25 μL. PCR amplification was performed with a 2720 Thermal Cycler (Applied Biosystems). PCR amplification conditions: 98 ℃ for 30 s, then perform 98 ℃ for 15 s, 46 ℃ for 30 s, 72 ℃ for 30 s, for a total of 32 cycles, extend for 5 min after 72 ℃, and store at 4 ℃. Shanghai Personal Biotechnology Co performed the PCR amplification and sequencing work.

### Bioinformatics analysis

The resulting sequences were spliced and dereplication using Vsearch (v2.13.4_linux_x86_64) and cutadapt (v2.3). After processing, the obtained high-quality sequences were divided into OTUs [58] with a threshold of 97%. The OTU information was obtained using Qiime2(2019.4) compared to the National center for biotechnology information database. Alpha diversity of microbiota was evaluated using Chao1, Simpson, Shannon, Observed specie (observed OTUs), Pielous evenness, Goods coverage index and rank abundance curve. Beta diversity of the microbiota was evaluated by PCoA and Adonis test. The above analyses were calculated from OTUs data using R language and visualized.

### Statistical analysis

Statistical analysis was performed using IBM SPSS (v25.0). Experimental results data were expressed as mean ± standard deviation, and independent t-test or Wilcoxon rank-sum test was used depending on whether the data were normally distributed and the variance was consistent. *P* < 0.05 was considered a significant difference [54].

## Data Availability

All data generated or analyzed during this study were included in this article. The metagenome raw sequence dataset has been uploaded to the NCBI database under accession number PRJNA799679 (https://www.ncbi.nlm.nih.gov/Traces/study/?acc=PRJNA799679&o=acc_s%3Aa).

## Abbreviations

HFHPD: High-fat and high-protein diet
OTU: Operational taxonomic unit
*C. difficile*: *Clostridioides difficile*
AAD: Antibiotic-associated diarrhea
PCoA: Principal coordinates analysis
PBS: Phosphate buffer solution
SDS: Sodium dodecyl sulfate
CTAB: Cetyl trimethyl ammonium bromide
